# Vitellogenins Are New High Molecular Weight Components and Allergens (Api m 12 and Ves v 6) of *Apis mellifera* and *Vespula vulgaris* Venom

**DOI:** 10.1371/journal.pone.0062009

**Published:** 2013-04-23

**Authors:** Simon Blank, Henning Seismann, Mareike McIntyre, Markus Ollert, Sara Wolf, Frank I. Bantleon, Edzard Spillner

**Affiliations:** 1 Institute of Biochemistry and Molecular Biology, University of Hamburg, Hamburg, Germany; 2 Clinical Research Division of Molecular and Clinical Allergotoxicology, Department of Dermatology and Allergy Biederstein, Technische Universität München, Munich, Germany; NIAID, United States of America

## Abstract

**Background/Objectives:**

Anaphylaxis due to hymenoptera stings is one of the most severe clinical outcomes of IgE-mediated hypersensitivity reactions. Although allergic reactions to hymenoptera stings are often considered as a general model for the underlying principles of allergic disease, venom immunotherapy is still hampered by severe systemic side effects and incomplete protection. The identification and detailed characterization of all allergens of hymenoptera venoms might result in an improvement in this field and promote the detailed understanding of the allergological mechanism. Our aim was the identification and detailed immunochemical and allergological characterization of the low abundant IgE-reactive 200 kDa proteins of *Apis mellifera* and *Vespula vulgaris* venom.

**Methods/Principal Findings:**

Tandem mass spectrometry-based sequencing of a 200 kDa venom protein yielded peptides that could be assigned to honeybee vitellogenin. The coding regions of the honeybee protein as well as of the homologue from yellow jacket venom were cloned from venom gland cDNA. The newly identified 200 kDa proteins share a sequence identity on protein level of 40% and belong to the family of vitellogenins, present in all oviparous animals, and are the first vitellogenins identified as components of venom. Both vitellogenins could be recombinantly produced as soluble proteins in insect cells and assessed for their specific IgE reactivity. The particular vitellogenins were recognized by approximately 40% of sera of venom-allergic patients even in the absence of cross-reactive carbohydrate determinants.

**Conclusion:**

With the vitellogenins of *Apis mellifera* and *Vespula vulgaris* venom a new homologous pair of venom allergens was identified and becomes available for future applications. Due to their allergenic properties the honeybee and the yellow jacket venom vitellogenin were designated as allergens Api m 12 and Ves v 6, respectively.

## Introduction

Systemic IgE-mediated allergic reactions after insect stings are prevalent causes of life-threatening and sometimes fatal immune-mediated anaphylaxis in humans. Although venom immunotherapy (VIT) is effective in the majority of patients the occurrence of systemic side effects in 20–40% of treated individuals and the failure of treatment in 10–20% of patients with honeybee venom (HBV) allergy [1], [2] demand a component-resolved approach to hymenoptera venom allergy. Since the use of native allergens is often hampered by means of quantity and purity recombinant allergens are increasingly introduced into diagnostic and therapeutic applications [3]. Moreover, the recombinant availability is a prerequisite for the rational design of hypoallergenic variants and molecules with defined characteristics such as proper folding and glycosylation and concurrent lack of cross-reactive carbohydrate determinants (CCDs) which still represent a challenge for adequate allergy diagnosis and the identification of clinically relevant allergens. Although in the field of hymenoptera venom allergy recently recombinant marker allergens have become commercially available for diagnostic purposes that have lead to an improvement [4]–[6], only a limited number of venom allergens is available as recombinant proteins.

Among the best characterized HBV allergens are phospholipase A2 (Api m 1), hyaluronidase (Api m 2), acid phosphatase (Api m 3), and the basic peptide melittin (Api m 4) all constituting medium to higher abundance proteins [7], [8]. Prominent yellow jacket venom (YJV) allergens include phospholipase A1 (Ves v 1), hyaluronidase (Ves v 2) for that recently a second isoform was identified [9], and antigen 5 (Ves v 5) [10], [11]. Api m 1 [12], [13] and Api m 2 [14]–[16] as well as Ves v 1 [17], [18], Ves v 2 [16], [19], and Ves v 5 [18], [20] could be expressed in bacteria, yeast or baculovirus-infected insect cells and selected structures were elucidated [19], [21], [22]. Recently the acid phosphatase of bee venom was cloned and recombinantly expressed [23] and with the dipeptidylpeptidase enzymes allergen C (Api m 5) and its vespid homologue Ves v 3, we could describe a novel class of hymenoptera venom enzymes [24]. Moreover, we could demonstrate that the lower abundance allergen Api m 10 is a major HBV allergen of considerable interest for diagnostic as well as therapeutic purposes [25] and also the Major Royal Jelly proteins 8 and 9 (Api m 11) could be characterized in detail applying recombinant strategies [26].

Due to the fact that all of the components in the venom may contribute to sensitization, symptoms, and success of VIT, their detailed characterization is of considerable interest. Moreover, insect venoms represent an interesting model system for allergic reactions since a relatively pure and more or less defined cocktail is injected into the patient.

In this study, we report the identification and molecular cloning of the 200 kDa high molecular weight allergens Api m 12 and Ves v 6 of *A. mellifera* and *V. vulgaris* venom which are present as prominent bands in Western blots with sera of venom-allergic patients, their recombinant production in insect cells and their detailed immunochemical characterization. Both allergens belong to the family of vitellogenins, present in most oviparous animals, and represent the first vitellogenin allergens identified in hymenoptera venoms. Sensitization to Api m 12 and Ves v 6 without interference of cross-reactive carbohydrate determinants in a population of HBV- and YJV-allergic patients was addressed by specific IgE (sIgE) measurement. The obtained data suggest a relevant role for Api m 12 and Ves v 6 as sensitizing venom component and as a novel cross-reactive class of homologous allergens in hymenoptera venoms potentially responsible for double positive test results with HBV and YJV apart from CCDs.

## Methods

### Materials

Crude honeybee venom was purchased from Latoxan (Valence, France). Anti-V5 antibody was purchased from Invitrogen (Karlsruhe, Germany). Polyclonal rabbit anti-HRP serum as well as anti-rabbit-IgG alkaline phosphatase (AP) conjugate and anti-mouse IgG AP conjugate were obtained from Sigma (Taufkirchen, Germany). The monoclonal AP conjugated anti-IgE antibody was purchased from BD Pharmingen (Heidelberg, Germany). AlaBlots™ were obtained from Siemens Healthcare Diagnostics (Los Angeles, Ca).

### Ethics Statement/Patients

Sera from venom-sensitized patients with HBV- and/or YJV-specific IgE and/or positive intradermal skin test results were collected during daily clinical practice. The detailed description of the characterization of venom-allergic patients as well as the serological patient data are depicted in the supplementary data table S1. All patients had given their informed written consent to draw an additional serum sample and all experiments applying human sera were approved by the local ethics committee of the faculty of medicine of the Technische Universität München, Munich, Germany.

### Protein Biochemistry

400 µg of *A. mellifera* venom dissolved in 30 µl 5x PAGE loading dye were subjected to SDS-PAGE. Bands of 200 kDa were excised, the proteins digested in-gel by trypsin (Roche Diagnostics, Penzberg, Germany) and resulting peptide fragments were sequenced on a Waters Micromass QToF2 mass spectrometer (Waters, Milford, MA, USA) by tandem mass spectrometry (MS/MS) according to the manufacturer’s instructions.

### Cloning of cDNA

Total RNA was isolated from the separated stinger with attached venom sack and additional glands from honeybee (*Apis mellifera*) and yellow jacket (*Vespula vulgaris*) using peqGold TriFast™ (Peqlab Biotechnologie, Erlangen, Germany). SuperScript III Reverse Transcriptase (Invitrogen) and the honeybee gene-specific primers 5′-TTAAGCCTTGCAAACGAAAGGAACGGTC-3′ and 5‘-CCAGAGGAACGAGCTCTTCGGGGAC-3′ and an oligo-dT_24_ primer in the case of *V. vulgaris* were used to synthesize cDNA from the isolated total RNA. First strand cDNA was used as template for PCR amplification of the coding sequences.

The Api m 12 mature protein coding sequence was amplified as two overlapping fragments using Phusion High Fidelity Master mix (New England Biolabs, Frankfurt, Germany). The N-terminal part was amplified using the forward primer 5′-GATCTCTAGAGCCGACTTCCAGCACAATTGGCAAGTCG-3′, adding an XbaI restriction site and the reverse primer 5‘-GATCCTGCAGGCGGCCGCCCAGAGGAACGAGCTCTTCGGGGAC-3′, adding a PstI as well as a NotI restriction site. The C-terminal fragment was amplified using the forward primer 5′-AGCCTGAGGAGCGTGAAGGACCG-3′ and the reverse primer 5′-GATCGCGGCCGCTTAAGCCTTGCAAACGAAAGGAACGGTC-3′, adding a NotI restriction site. Afterwards, the N-terminal fragment was subcloned via XbaI and PstI into the vector pUC19 (Roth, Karlsruhe, Germany). After verification of the sequence the C-terminal fragment was cloned via Bsu36I, present in the overlapping sequence of both Api m 12 fragments and NotI into the pUC19 containing the N-terminal fragment. The resulting full length coding region was cut out via XbaI and NotI and subcloned into the digested baculovirus transfer vector pAcGP67B (BD Pharmingen, Heidelberg, Germany) which was modified by addition of an N-terminal 10-fold His-tag, V5 epitope as well as an XbaI restriction site.

Due to the lack of genomic data for *Vespula vulgaris* a C-terminal part of Ves v 6 was amplified from venom gland cDNA using two degenerate forward primers corresponding to the conserved GL/ICG motif (5′-GGICT(GC)TG(CT)GG-3′ and 5′-GGIAT(CT)TG(CT)GG-3′) as described by Lee et al. [27] and oligodT_24_ back primer. After sequence determination of subcloned cDNA fragments the information was used as basis for further sequence determination by 5′RACE employing the 5′/3′RACE Kit, Second Generation (Roche, Grenzach, Germany) according to the recommendations of the manufacturer.

The Ves v 6 mature protein coding sequence was then amplified as two overlapping fragments using Phusion High Fidelity Master mix. The C-terminal part was amplified using the forward primer 5′-GATCCTCGAGAAGTGGGAAGATATGTTGTCCCCG-3′, adding an XhoI restriction site and the reverse primer 5‘-GATCGCGGCCGCTCAGGTAGCTACACATTCG-3′, adding a NotI restriction site. The N-terminal part was amplified using the forward primer 5′-GATCCTCGAGGATAACAACATCGAGCATGGCTGG-3′, adding an XhoI restriction site and the reverse primer 5′-CTTGTAGGTCAGGCTTGACAAAG-3′. Afterwards, the C-terminal part was subcloned via XhoI and NotI into the vector pFastBac1 (Invitrogen). After verification of the sequence the N-terminal fragment was cloned via Bsu36I, present in the overlapping sequence of both Ves v 6 fragments and XhoI into pFastBac1 containing the C-terminal fragment. After transformation of DH10-Bac cells (Invitrogen) the bacmid-DNA was isolated from overnight cultures of white colonies according to the recommendations of the manufacturer.

### Recombinant Baculovirus Production

*Spodoptera frugiperda* cells (Sf9) (Invitrogen) were grown at 27°C in serum-free medium (Lonza, Verviers, Belgium) containing 10 µg/ml gentamycin (Invitrogen). Cell density was determined by haemocytometer counts, cell viability was evaluated by staining with Trypan Blue. In the case of Api m 12 recombinant baculovirus was generated by cotransfection of Sf9 cells with BaculoGold bright DNA (BD Pharmingen) and the baculovirus transfer vector pAC-GP67-B containing Api m 12. Recombinant Ves v 6 baculovirus was generated by transfection of Sf9 cells with the recombinant bacmid-DNA according to the recommendations of the manufacturer.

High titer stocks were produced by three rounds of virus amplification and optimal MOI for protein expression was determined empirically by infection of Sf9 cells in 100 ml suspension flasks (1.5×10^6^ cells/ml in 20 ml suspension culture) with serial dilutions of high titer virus stock.

### Expression in Baculovirus-infected Insect Cells

High titer stocks of recombinant baculovirus were used to infect 400 ml suspension cultures of Sf9 cells (1.5×10^6^ cells per ml) in 2000 ml flasks. For protein production the cells were incubated at 27°C and 110 rpm for 72 h.

### Protein Purification

The supernatant of baculovirus-infected cells was collected, adjusted to pH 8, centrifuged at 4000×g for 5 minutes, and applied to a nickel-chelating affinity matrix (NTA-agarose, Qiagen, Hilden, Germany). The column was washed with binding buffer (50 mM sodium phosphate, pH 7.6, 500 mM NaCl) and pre-eluted with binding buffer containing 20 mM imidazole. The recombinant protein was eluted from the matrix using binding buffer containing 300 mM imidazole. Purification was confirmed by SDS-PAGE.

### Western Blotting

For immunoblot procedures the purified recombinant allergens were separated by SDS-PAGE and immobilized onto nitrocellulose membranes. For Western blot procedures with anti-V5 epitope mAb the antibody was applied according to the recommendations of the manufacturer and bound antibodies visualized via anti-mouse IgG AP conjugate and nitrotetrazolium blue chloride/5-bromo-4-chloro-3-indoyl phosphate according to the recommendations of the manufacturer. Lectin blots (DIG Glycan differentiation Kit, Roche Diagnostics, Mannheim, Germany) were performed according to the recommendations of the manufacturer.

### Immunoreactivity of Patient Sera with Recombinant Proteins

For assessment of specific IgE immunoreactivity of human sera in ELISA, 384 well microtiter plates (Greiner, Frickenhausen, Germany) were coated with purified recombinant proteins (20 µg/ml) at 4°C overnight and blocked with 40 mg/ml milkpowder in PBS. Thereafter, human sera were diluted 1∶2 with PBS and incubated in a final volume of 20 µl for 4 hours at room temperature. After washing 4 times with PBS bound IgE were detected with a monoclonal AP-conjugated anti-human IgE antibody diluted 1∶1000. After washing 4 times with PBS 50 µl of substrate solution (5 mg/ml 4-nitrophenylphosphate, AppliChem, Darmstadt, Germany) per well were added. The plates were read at 405 nm. The lower end functional cut-off indicated as lines was calculated as the mean of the negative controls plus 2 standard deviations (SD). Reactivities only slightly higher than the cut-off were excluded. For ELISA procedures with anti-V5 epitope mAb and anti-HRP antiserum the antibodies were applied according to the recommendations of the manufacturer and bound antibodies visualized via corresponding secondary antibodies conjugated to AP as described above.

### Other Methods

Molecular biology standard procedures such as PCR, DNA-restriction, ligation, transformation, and plasmid-isolation were performed according to established protocols [28].

## Results

### Identification of Vitellogenin in Honeybee and Yellow Jacket Venom

**Figure 1:**
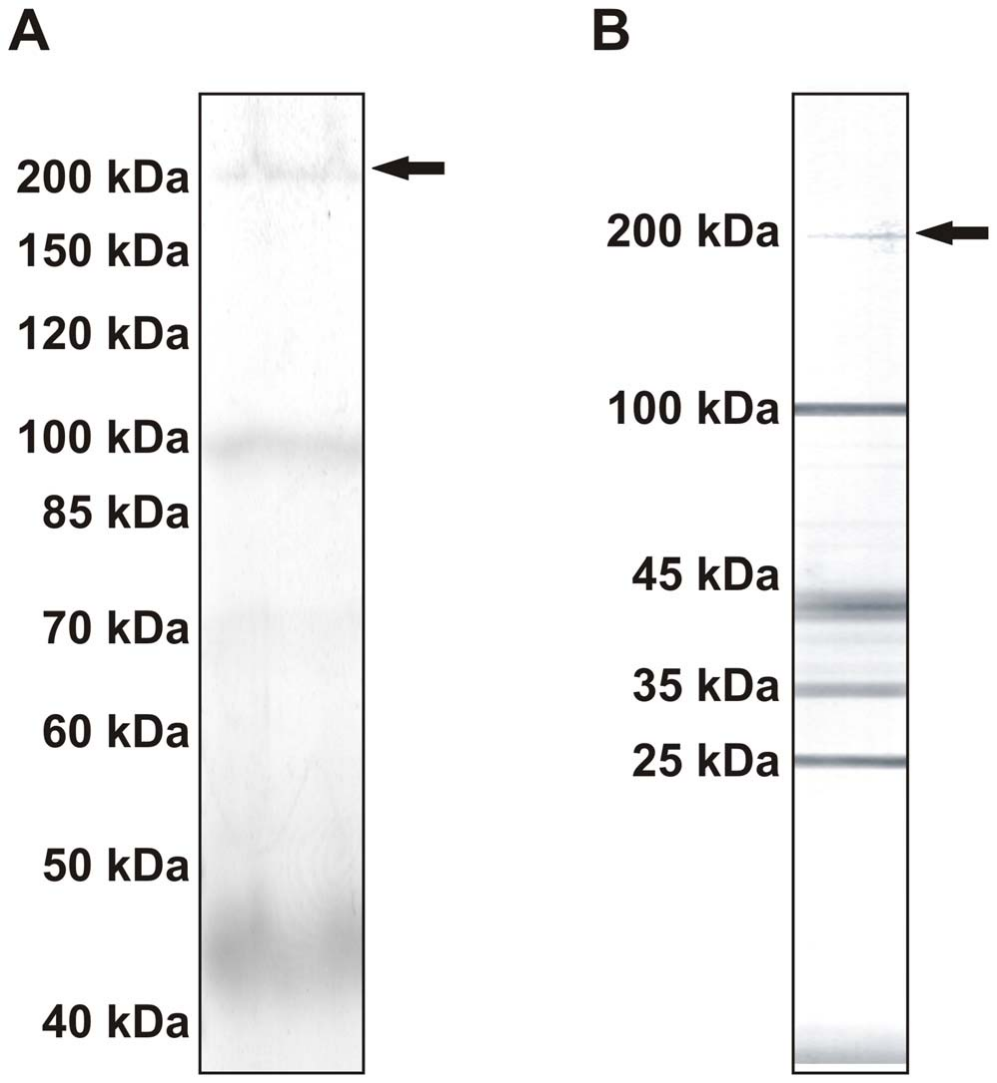
Detection of Api m 12 and Ves v 6 in *A. mellifera* and *V. vulgaris* venom. A SDS-PAGE and Coomassie blue staining of the high molecular weight fraction of honeybee venom. The arrow indicates the 200 kDa band that was subjected to MS/MS-based sequencing. B IgE Immunoreactivity of pooled sera from YJV-sensitized patients with the venom of *V. vulgaris* in Western Blot (AlaBlot™). The arrow indicates the reactive 200 kDa protein band.

Both, honeybee and yellow jacket venom contain components of 200 kDa with unknown identity (Fig. 1). Moreover, immunoblots of *Vespula vulgaris* venom (AlaBlots™) with YJV allergic patient sera show specific IgE reactivity in the range of 200 kDa (Fig. 1B). A corresponding reactivity in honeybee AlaBlots™ was not detected due to another electrophoretic separation and the missing of this molecular weight range. In order to identify the honeybee venom protein, the 200 kDa band of *A. mellifera* venom (Fig. 1A) was subjected to sequencing by tandem mass spectrometry.

Sequencing of the protein yielded 5 tryptic fragments that could be assigned to vitellogenin (Genbank accession NP_001011578), a protein consisting of 1770 amino acids, with a signal peptide of 16 amino acids and a calculated molecular weight of 201 kDa.

On the basis of this information and due to the lack of genomic sequence information the full coding region of the *V. vulgaris* vitellogenin was identified from venom gland cDNA by PCR- and 5′-RACE-based strategies. The 3′ region was obtained using degenerate primers designed against the GL/ICG motif which is conserved among all insect vitellogenins [27] and oligo-dT_24_ back primer and the 5′ region was cloned applying the 5′RACE strategy.

**Figure 2:**
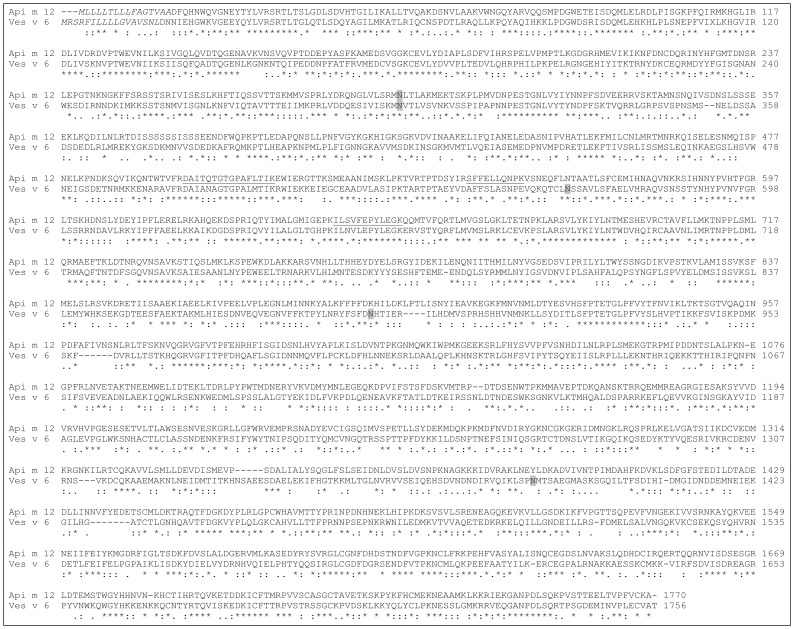
Alignment of Api m 12 and Ves v 6. Alignment of *A. mellifera* vitellogenin Api m 12 (Genbank accession NP_001011578) and *V. vulgaris* vitellogenin Ves v 6 (Genbank accession AER70365) reveals an identity of 40% on protein level. Asterisks, colons, and periods indicate fully conserved, strongly similar, and weakly similar residues, respectively. Peptides identified by mass spectrometry are underlined, signal sequences are italicized, and putative N-glycosylation sites are highlighted in grey.

**Figure 3:**
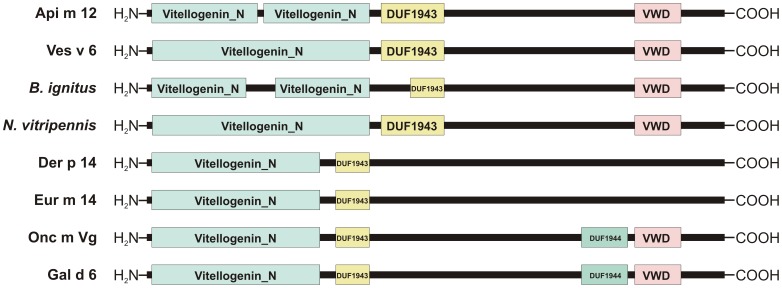
Domain architecture of Api m 12, Ves v 6 and other vitellogenins. Comparison of the domain architecture of Api m 12 and Ves v 6 with that of the vitellogenins from the hymenoptera species *Bombus ignitus* (Genbank accession ACM46019) and *Nasonia vitripennis* (Genbank accession XP_001607388) as well as with that of vitellogenin allergens of the mites *Dermatophagoides pteronyssinus* (Der p 14, Genbank accession AAM21322) and *Euroglyphus maynei* (Eur m 14, Genbank accession AAF14270), the fish *Oncorhynchus mykiss* (Onc m Vg, Genbank accession CAA63421), and *Gallus gallus* (Gal d 6, Genbank accession AAA49139). DUF, domain of unknown function; VWD, von Willebrand factor type D domain.

The identified full coding region of *V. vulgaris* vitellogenin (Genbank accession JN794080) consists of 1756 amino acids with a signal peptide cleavage site between amino acids 17 and 18 and a calculated molecular weight of 199,7 kDa. The sequence identity with the honeybee vitellogenin is in the range of 40% on protein level (Fig. 2). Moreover the yellow jacket and the honeybee vitellogenin contain 4 and 1 putative N-glycosylation sites, respectively. The yellow jacket vitellogenin contains a conserved GLCG motif at position 1582–1585 which is found in honeybee vitellogenin at position 1596–1599 [29] followed by nine cysteines at in hymenoptera conserved positions near the C-terminus. In contrast to most other insect vitellogenins the vitellogenin of *V. vulgaris* lacks a DGXR motif upstream the GLCG motif. The honeybee protein consists of two N-terminal Vitellogenin_N multi-domains (each contains a Lipoprotein N-terminal Domain; LPD_N) separated by a short polyserine region followed by a domain of unknown function 1943 (DUF1943) and a von Willebrand factor type D domain (VWD) (Fig. 3). In contrast the *V. vulgaris* vitellogenin shows one big N-terminal Vitellogenin_N multi-domain that comprises the two Lipoprotein N-terminal domains followed by the DUF1932 and the VWD. The predicted vitellogenin from the wasp *Nasonia vitripennis* shows the same domain architecture when compared with that of *V. vulgaris* and the vitellogenin from the bumblebee *Bombus ignitus* a similar domain structure as the honeybee protein with the exception that the Vitellogenin_N domains as well as the DUF1943 are shorter.

Additionally figure 3 shows the domain architecture of other vitellogenin allergens from mites, fish and chicken egg all of which contain only one N-terminal Vitellogenin_N domain (LPD_N) followed by a short DUF1943. The vitellogenin allergens from the mites *Dermatophagoides pteronyssinus* and *Euroglyphus maynei* are devoid of a von Willebrand factor type D domain whereas in the vitellogenin allergens from fish (*Oncorhynchus mykiss*) and chicken (*Gallus gallus*) the VWD is headed by a second domain of unknown function (DUF1944) not present in the other depicted vitellogenins.

According to their presence in the venom and their allergenic properties (see below) the vitellogenins of *A. mellifera* and *V. vulgaris* were designated as allergens Api m 12 and Ves v 6, respectively, by the Allergen Nomenclature Sub-Committee of the International Union of Immunology Societies (IUIS).

### Recombinant Expression of Api m 12 and Ves v 6

**Figure 4:**
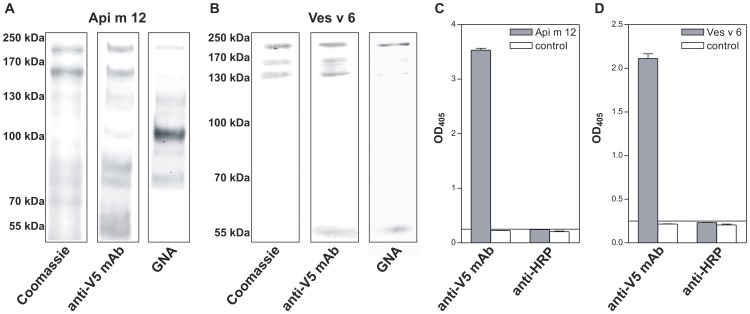
Recombinant expression and immunoreactivity of Api m 12 and Ves v 6. A, B SDS-PAGE and immunoblot analyses of Api m 12 and Ves v 6 recombinantly produced in Sf9 insect cells visualized by Coomassie blue staining, monoclonal anti-V5 epitope antibody and *Galanthus nivalis* agglutinin (GNA), recognizing terminal mannose 1,2-, 1,3-, and 1,6-linked to mannose. C, D Immunoreactivity of recombinant Api m 12 and Ves v 6 in ELISA using the monoclonal anti-V5 epitope antibody and polyclonal anti-HRP antiserum specific for α1,3-fucose residues, the underlying principle of hymenoptera venom cross-reactive carbohydrate determinant (CCD) reactivity.

For assessment of the immunoreactivity of Api m 12 and Ves v 6 both mature vitellogenins were recombinantly produced as secreted full-length proteins by baculovirus-mediated infection of Sf9 (*Spodoptera frugiperda*) insect cells. Supernatants of expression cultures were subjected to Ni-affinity chromatography and the resulting proteins analyzed by SDS-PAGE, Western blotting and ELISA (Fig. 4).

Both proteins were obtained in soluble and secreted form with yields of approximately 0.1 µg/ml of culture supernatant. As expected, recombinant Api m 12 migrated as 200 kDa band in SDS-PAGE but showed additional bands of lower molecular weight (Fig. 4A), apparently all of which proved to be reactive with IgE antibodies of venom allergic patients (Fig. S1). The molecular size of the fragments however renders IgE reactivity likely. The reactivity of these bands with a monoclonal V5 epitope tag antibody confirmed the identity of the recombinant protein bands (Fig. 4A, middle). Similarly, recombinant yellow jacket Ves v 6 migrated as three visible bands reactive with the V5 epitope antibody with molecular weights of approximately 200, 160, and 130 kDa (Fig. 4B). It is reported that honeybee vitellogenin is subject of further processing [30] so that it has to be speculated that the bands detected below 200 kDa represent processed molecules.

In order to analyze if the recombinant vitellogenins are glycosylated lectin blots were performed. *Galanthus nivalis* agglutinin (GNA) recognizes terminal mannose 1,2-, 1,3- or 1,6-linked to mannose, a structure which is present in native as well as in recombinant in lepidopteran insect cells produced hymenoptera venom allergens. Thus, GNA reactivity indicates the presence of N-linked glycans. In immunoblot analyses of Api m 12 GNA reacted with the 200 kDa band as well as with the lower molecular weight products above approximately 80 kDa whereby the strongest reactivity was observed with a band of approximately 100 kDa (Fig. 4A, right). For recombinant Ves v 6 all bands proved to be glycosylated (Fig. 4B, right) a fact that might be due to the presence of more putative glycosylation sites when compared to Api m 12. Api m 12 carries 3 potential N-glycosylation sites but only one of them it is likely to be glycosylated according to NetNGlyc Prediction server. In contrast Ves v 6 contains 4 sites in its sequence with a high probability to be glycosylated.

Although Api m 12 and Ves v 6 proved to be glycosylated the missing reactivity of both with anti-HRP rabbit serum, specific for alpha1,3-core fucosylation of N-glycans, the structure responsible for CCD-based cross-reactivity, demonstrated the lack of cross-reactive carbohydrate determinants (Fig. 4C and D) as shown previously for other insect venom allergens produced in Sf9 insect cells [13], [16], [25], [26].

Taken together, these data demonstrate for the first time that insect cells are suitable hosts for the production of vitellogenins. Moreover Api m 12 and Ves v 6 produced in Sf9 cells represent adequate molecules to assess their relevance as proteinogenic allergens beyond carbohydrate-based cross-reactivity.

### IgE Immunoreactivity of Patient Sera with Recombinant Api m 12 and Ves v 6

To evaluate the IgE immunoreactivity of Api m 12 and Ves v 6 produced in Sf9 cells, individual sera of patients with a clinical history of an allergic reaction after a stinging event (Table S1) were analyzed by ELISA for specific IgE antibodies. All patients were recruited during daily clinical practice and had sIgE for HBV (i1) and/or YJV (i3) and/or showed a positive intradermal skin test with HBV and/or YJV.

**Figure 5:**
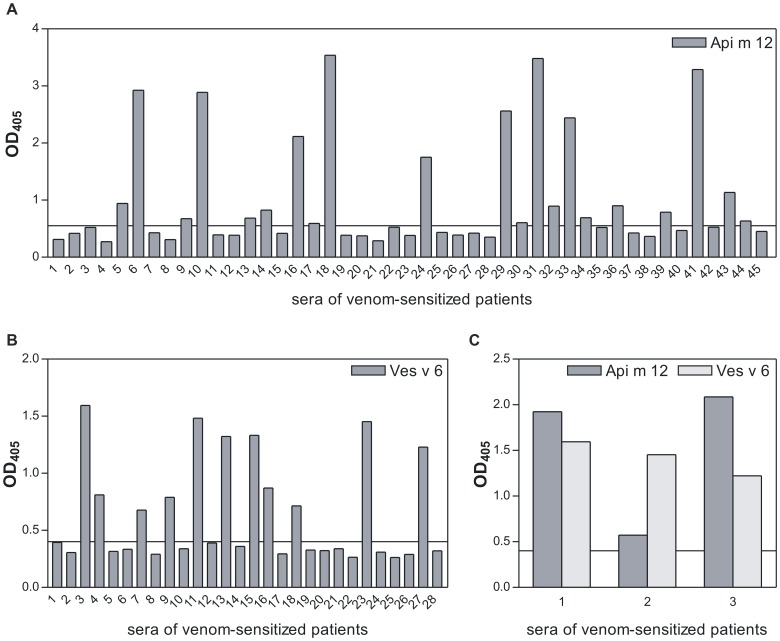
IgE immunoreactivity of individual patient sera with recombinant Api m 12 and Ves v 6. A IgE reactivity of individual sera from HBV-sensitized patients with Api m 12 in ELISA. B IgE reactivity of individual sera from YJV-sensitized patients with Ves v 6 in ELISA. C IgE reactivity of exemplary sera from patients that show a monosensitization to either HBV or YJV in intradermal skin test with Api m 12 and Ves v 6 in ELISA. Sera 1 and 2 correspond to sera 3 and 23 in figure 5B and serum 3 to serum 41 in figure 5A. The lower end functional cut-off of the ELISAs is represented as solid line.

Of the 45 patients with positive test to HBV 20 (44%) showed specific IgE reactivity with Api m 12 (Fig. 5A) and of the 28 patients with positive test to YJV 11 (39%) showed specific IgE reactivity with Ves v 6 (Fig. 5B). Since allergens produced in Sf9 insect cells are devoid of CCD reactivity these reactivities can be attributed to proteinous epitopes exclusively.

Api m 12 and Ves v 6 share a sequence identity of approximately 40% on protein level so that a presence of shared epitopes and hence of cross-reactivity on protein level is likely. To test this hypothesis we analyzed sera from patients either negative with HBV or YJV in intradermal skin test for sIgE reactivity with Api m 12 and Ves v 6 (Fig. 5C). The patients 1 and 2 showed negative results in skin test with HBV and positive results with YJV and patient 3 vice versa. The reactivity of all three sera with both, Api m 12 and Ves v 6 strongly suggests molecular cross-reactivity on the protein level or at least co-sensitization beyond cross-reactive carbohydrate determinants.However, not all sera reacting with one of the allergens showed cross-reactivity with the other one (data not shown).

These data demonstrate that the high molecular weight allergens Api m 12 and Ves v 6 are components of honeybee and yellow jacket venom with IgE-sensitizing potential in approximately 40% of venom-allergic patients beyond carbohydrate-based reactivity. This finding renders the vitellogenins important allergens. Moreover the newly identified vitellogenins represent a novel pair of cross-reactive pan-allergens in the venoms of *A. mellifera* and *V. vulgaris*.

## Discussion

In this study, we have identified and characterized the 200 kDa high molecular weight allergens in the venoms of the hymenoptera species *A. mellifera* and *V. vulgaris*, both belonging to the family of vitellogenins. Using advanced sequencing strategies to overcome quantity limitations we obtained sequence information of Api m 12 allowing assignment to honeybee vitellogenin [29]. Finally, we were able to amplify the full coding sequence from venom gland cDNA. On the basis of the obtained sequence information of Api m 12 as well as of the sequences of the homologues from *Bombus ignitus*, *B. hypocrita* [31] and *Nasonia vitripennis* we additionally were able to identify the corresponding protein Ves v 6 as a new allergen of *V. vulgaris* venom. This protein corresponds to Api m 12 in terms of molecular weight, amino acid sequence and IgE immunoreactivity.

Sequence analysis provides clear evidence that Api m 12 and Ves v 6 belong to the class of vitellogenins which are produced by most oviparous animals, both invertebrates and vertebrates, where after synthesis they are transported into oocytes to serve as food for the embryos [30]. Vitellogenins of invertebrate and vertebrate species represent a multigene superfamily together with insect apolipophorin II/I, human apolipoprotein B, and mammalian microsomal triglyceride transfer proteins [32]. In insects vitellogenin is produced in large amounts in the fat body, released in the hemolymph, and taken up by developing oocytes [33], [34]. Honeybee vitellogenin was first purified from hemolymph more than 20 years ago [35] and the cDNA cloned and sequenced in 2003 [29]. The largest amount of vitellogenin is found in the hemolymph of honeybee queens but it is also present in workers [36], [37]. Although it is often described as female-specific protein [38], it was identified in honeybee drones [31]. Honeybee vitellogenin is thought to act as multifunctional molecule that is involved in a vast number of processes such as hormone signalling, food-related behaviour, immunity, stress resistance, and longevity [39]–[43]. Although insect vitellogenins seem to be implicated in the transport of sugars, lipids, phosphates, vitamins and hormones [44], [45] it is largely unknown how the honeybee vitellogenin molecule exerts its many functions [30]. To date a plethora of data concerning honeybee vitellogenin is available, however, until now it never was identified as component of the venom. Although the function of vitellogenin is not restricted to reproduction all assumptions about its role as venom protein remain speculative. Nevertheless, the additional identification of vitellogenin as a component of vespid venom suggests the conclusion of a functional role.

The amplification of *A. mellifera* and *V. vulgaris* vitellogenin from venom gland cDNA allows the presumption that it is directly synthesized in the venom gland and secreted into the venom and does not stem from the fat body and is taken up by receptor-mediated endocytosis as described for the transport of vitellogenin to the ovaries [32]. To the best of our knowledge this is the first report describing the identification of a vitellogenin from a *Vespula* species which might help shedding light on the diverse functions of this protein family. Together with the vitellogenins from mites, fish and chicken egg the newly identified proteins represent a novel class of pan-allergens.

For the recombinant production of complex high molecular weight hymenoptera venom allergens insect cells appear the most appropriate system which are superior in terms of functionality, epitope authenticity, and folding [15], [16], [24]. The hallmark of CCDs of hymenoptera venom allergens are carbohydrates carrying α1,3-linked core L-fucose residues. IgE with specificity for CCDs play a key role in allergen- cross-reactivity, representing a major concern for the specificity of diagnostic approaches in hymenoptera venom allergy [16], [46]–[48]. We recently demonstrated that the use of Sf9 (*Spodoptera frugiperda*) insect cells for allergen expression represents a strategy to circumvent the establishment of CCDs while maintaining the advantages of an autologous eukaryotic expression system [13], [16], [25], [26].

Expression of both Api m 12 and Ves v 6 in Sf9 cells yielded soluble proteins with a molecular weight of 200 kDa but additionally products of lower molecular weight. These lower molecular weight products might be results of degradation. More likely however is that they represent further processed products. Functional honeybee vitellogenin from fat body is described to be further cleaved into a 40 kDa and a 150 kDa fragment and that the fragmentation pattern differs in fat body and hemolymph [30]. Both honeybee and vespid vitellogenin contain several putative cleavage sites showing a RXXR/S consensus sequence [45] which might be the reason for the fragmentation pattern observed for the recombinant proteins. Moreover, applying *Galanthus nivalis* agglutinin, recognizing terminal mannose residues, showed that Api m 12 and Ves v 6 from Sf9 cells are indeed glycosylated while the missing reactivity with an antiserum raised against horseradish peroxidase and specific for α1,3-core fucose proved that both molecules are devoid of any immunologically detectable CCD reactivity.

Applying the CCD-free recombinant Api m 12 and Ves v 6 in ELISA 44% and 39% of HBV- and YJV-sensitized patients, respectively proved to have specific IgE antibodies. The obtained data suggest that Api m 12 and Ves v 6 have to be considered as relevant venom allergens with IgE-sensitizing potential beyond CCDs. Interestingly, vitellogenins are also described as allergens of several other allergen sources including mites [49]–[51], fish eggs [52], [53], and chicken egg yolk [54]. Since sequence similarity with Api m 12 and Ves v 6 is rather low, the presence of cross-reactive epitopes is not very likely.

In contrast, Api m 12 and Ves v 6 share an identity of 40% on protein level and the exemplary reactivity analysis of sera from patients who show a monosensitization to HBV or YJV in intradermal skin test with both molecules suggests that the vitellogenins represent a novel pair of cross-reactive allergens in hymenoptera venom. Apart from CCDs so far, double-positivity in venom allergic patients had been largely attributed to IgE directed against either hyaluronidases (Api m 1 and Ves v 2) [55] or dipeptidylpeptidases (Api m 5 and Ves v 3) [24], although recent studies indicate that the cross-reactivity between the hyaluronidases on protein level is limited [16], [56] what is due to the absence of surface areas that possess a significant degree of identity [19]. However, since only a fraction of the analysed sera showed cross-reactivity with Api m 12 and Ves v 6 further studies should address the extend of epitope-based cross-reactivity.

In summary, we have identified the vitellogenins Api m 12 and Ves v 6 in the venom of *A. mellifera* and *V. vulgaris* as new allergens. Both allergens were cloned, produced by the baculovirus-mediated infection of Sf9 insect cells and assessed for their allergic potential. The obtained results clearly demonstrate an IgE-sensitizing potential beyond carbohydrate-based cross-reactivity in approximately 40% of venom-sensitized patients hinting for a role as relevant allergens in hymenoptera venom allergy. Moreover, Api m 12 and Ves v 6 represent the first vitellogenins identified in insect venoms and a new family of cross-reactive venom pan-allergens. Although the natural function of these multifunctional molecules in insect venoms is not known the recombinant availability of this new allergen pair allows for elucidation of individual component-resolved reactivity profiles and can provide insights into the role of particular venom components and therefore might contribute to a more detailed understanding of the molecular and allergological mechanisms of insect venoms.

## Supporting Information

Figure S1 **Immunoreactivity of Api m 12 with pooled sera of honeybee venom allergic patients.** Purified Api m 12 was separated by SDS-PAGE and immobilized onto a nitrocellulose membrane. Sera from 4 patients who showed specific IgE reactivity in ELISA (patients 16, 18, 24, and 29 in figure 5A) were pooled and diluted 1∶10 with 5 mg/ml BSA in PBS and applied to the Western blot. Visualization of bound IgE was then performed with anti-human IgE mAb conjugated to alkaline phosphatase and nitrotetrazolium blue chloride/5-bromo-4-chloro-3-indoyl phosphate.
(DOC)

Table S1 **Serological data of patients assessed in IgE reactivity analysis.** The sIgE levels for honeybee venom (HBV) (i1) and yellow jacket venom (YJV) (i3) were determined with the Immulite 2000 (Siemens Healthcare Diagnostics, Los Angeles, Ca.) or ImmunoCap 250 (Phadia, Uppsala, Sweden), and for Api m 12 and Ves v 6 as described for Fig. 5 (the lower end functional cutoff of the Api m 12 and Ves v 6 ELISA was OD_405_ = 0,55 and OD_405_ = 0,4, respectively). For intradermal testing of patients with suspected insect venom allergies serial 10-fold dilutions of venom extracts with concentrations ranging from 0.0001 to 0.1 mg/L were performed. Histamine hydrochloride and physiologic saline were used as positive and negative control solutions, respectively. Intradermal tests were rated positive when the wheal size was >5 mm in diameter with a surrounding erythema.
(DOC)

## References

[1] Muller U, Helbling A, Berchtold E (1992) Immunotherapy with honeybee venom and yellow jacket venom is different regarding efficacy and safety. J Allergy Clin Immunol 89: 529–535. 1740583 10.1016/0091-6749(92)90319-w

[2] Rueff F, Przybilla B, Muller U, Mosbech H (1996) The sting challenge test in Hymenoptera venom allergy. Position paper of the Subcommittee on Insect Venom Allergy of the European Academy of Allergology and Clinical Immunology. Allergy 51: 216–225. 8792917 10.1111/j.1398-9995.1996.tb04596.x

[3] Valenta R, Kraft D (2002) From allergen structure to new forms of allergen-specific immunotherapy. Curr Opin Immunol 14: 718–727. 12413521 10.1016/s0952-7915(02)00402-8

[4] Hofmann SC, Pfender N, Weckesser S, Huss-Marp J, Jakob T (2011) Added value of IgE detection to rApi m 1 and rVes v 5 in patients with Hymenoptera venom allergy. J Allergy Clin Immunol 127: 265–267. 20719373 10.1016/j.jaci.2010.06.042

[5] Hoffman SC, Pfender N, Weckesser S, Blank S, Huss-Marp J, et al (2011) Detection of IgE to a panel of species specific allergens further improves discrimination of bee and wasp venom allergy (Reply). J Allergy Clin Immunol 128: 248.21565392

[6] Korosec P, Valenta R, Mittermann I, Celesnik N, Silar M, et al (2012) High sensitivity of CAP-FEIA rVes v 5 and rVes v 1 for diagnosis of Vespula venom allergy. J Allergy Clin Immunol 129: 1406–1408. 22277201 10.1016/j.jaci.2011.12.975 PMC6624139

[7] Arbesman CE, Reisman RE, Wypych JI (1976) Allergenic potency of bee antigens measured by RAST inhibition. Clin Allergy 6: 587–595. 1016291 10.1111/j.1365-2222.1976.tb01945.x

[8] Müller UR (1988) Insektenstichallergie: Klinik, Diagnostik und Therapie. Stuttgart, New York: Gustav Fischer Verlag.

[9] Kolarich D, Leonard R, Hemmer W, Altmann F (2005) The N-glycans of yellow jacket venom hyaluronidases and the protein sequence of its major isoform in Vespula vulgaris. Febs J 272: 5182–5190. 16218950 10.1111/j.1742-4658.2005.04841.x

[10] King TP, Spangfort MD (2000) Structure and biology of stinging insect venom allergens. Int Arch Allergy Immunol 123: 99–106. 11060481 10.1159/000024440

[11] Muller UR (2002) Recombinant Hymenoptera venom allergens. Allergy 57: 570–576. 12100296 10.1034/j.1398-9995.2002.02157.x

[12] Dudler T, Chen WQ, Wang S, Schneider T, Annand RR, et al (1992) High-level expression in Escherichia coli and rapid purification of enzymatically active honey bee venom phospholipase A2. Biochim Biophys Acta 1165: 201–210. 1450215 10.1016/0005-2760(92)90188-2

[13] Blank S, Michel Y, Seismann H, Plum M, Greunke K, et al (2011) Evaluation of different glycoforms of honeybee venom major allergen phospholipase A2 (Api m 1) produced in insect cells. Protein Pept Lett 18: 415–422. 21171948 10.2174/092986611794653923

[14] Gmachl M, Kreil G (1993) Bee venom hyaluronidase is homologous to a membrane protein of mammalian sperm. Proc Natl Acad Sci U S A 90: 3569–3573. 7682712 10.1073/pnas.90.8.3569 PMC46342

[15] Soldatova LN, Crameri R, Gmachl M, Kemeny DM, Schmidt M, et al (1998) Superior biologic activity of the recombinant bee venom allergen hyaluronidase expressed in baculovirus-infected insect cells as compared with Escherichia coli. J Allergy Clin Immunol 101: 691–698. 9600508 10.1016/S0091-6749(98)70179-4

[16] Seismann H, Blank S, Braren I, Greunke K, Cifuentes L, et al (2010) Dissecting cross-reactivity in hymenoptera venom allergy by circumvention of alpha-1,3-core fucosylation. Mol Immunol 47: 799–808. 19896717 10.1016/j.molimm.2009.10.005

[17] King TP, Lu G, Gonzalez M, Qian N, Soldatova L (1996) Yellow jacket venom allergens, hyaluronidase and phospholipase: sequence similarity and antigenic cross-reactivity with their hornet and wasp homologs and possible implications for clinical allergy. J Allergy Clin Immunol 98: 588–600. 8828537 10.1016/s0091-6749(96)70093-3

[18] Seismann H, Blank S, Cifuentes L, Braren I, Bredehorst R, et al (2010) Recombinant phospholipase A1 (Ves v 1) from yellow jacket venom for improved diagnosis of hymenoptera venom hypersensitivity. Clin Mol Allergy 8: 7. 20359368 10.1186/1476-7961-8-7 PMC2867971

[19] Skov LK, Seppala U, Coen JJ, Crickmore N, King TP, et al (2006) Structure of recombinant Ves v 2 at 2.0 Angstrom resolution: structural analysis of an allergenic hyaluronidase from wasp venom. Acta Crystallogr D Biol Crystallogr 62: 595–604. 16699186 10.1107/S0907444906010687

[20] Henriksen A, King TP, Mirza O, Monsalve RI, Meno K, et al (2001) Major venom allergen of yellow jackets, Ves v 5: structural characterization of a pathogenesis-related protein superfamily. Proteins 45: 438–448. 11746691 10.1002/prot.1160

[21] Scott DL, Otwinowski Z, Gelb MH, Sigler PB (1990) Crystal structure of bee-venom phospholipase A2 in a complex with a transition-state analogue. Science 250: 1563–1566. 2274788 10.1126/science.2274788

[22] Markovic-Housley Z, Miglierini G, Soldatova L, Rizkallah PJ, Muller U, et al (2000) Crystal structure of hyaluronidase, a major allergen of bee venom. Structure 8: 1025–1035. 11080624 10.1016/s0969-2126(00)00511-6

[23] Grunwald T, Bockisch B, Spillner E, Ring J, Bredehorst R, et al (2006) Molecular cloning and expression in insect cells of honeybee venom allergen acid phosphatase (Api m 3). J Allergy Clin Immunol 117: 848–854. 16630944 10.1016/j.jaci.2005.12.1331

[24] Blank S, Seismann H, Bockisch B, Braren I, Cifuentes L, et al (2010) Identification, recombinant expression, and characterization of the 100 kDa high molecular weight hymenoptera venom allergens Api m 5 and Ves v 3. J Immunol 184: 5403–5413. 20348419 10.4049/jimmunol.0803709

[25] Blank S, Seismann H, Michel Y, McIntyre M, Cifuentes L, et al (2011) Api m 10, a genuine A. mellifera venom allergen, is clinically relevant but underrepresented in therapeutic extracts. Allergy 66: 1322–1329. 21658068 10.1111/j.1398-9995.2011.02667.x

[26] Blank S, Bantleon FI, McIntyre M, Ollert M, Spillner E (2012) The major royal jelly proteins 8 and 9 (Api m 11) are glycosylated components of Apis mellifera venom with allergenic potential beyond carbohydrate-based reactivity. Clin Exp Allergy 42: 976–985. 22909169 10.1111/j.1365-2222.2012.03966.x

[27] Lee JM, Hatakeyama M, Oishi K (2000) A simple and rapid method for cloning insect vitellogenin cDNAs. Insect Biochem Mol Biol 30: 189–194. 10732986 10.1016/s0965-1748(99)00127-7

[28] Ausubel FM (1996) Current Protocols in Molecular Biology. New York: Wiley Interscience.

[29] Piulachs MD, Guidugli KR, Barchuk AR, Cruz J, Simoes ZL, et al (2003) The vitellogenin of the honey bee, Apis mellifera: structural analysis of the cDNA and expression studies. Insect Biochem Mol Biol 33: 459–465. 12650694 10.1016/s0965-1748(03)00021-3

[30] Havukainen H, Halskau O, Skjaerven L, Smedal B, Amdam GV (2011) Deconstructing honeybee vitellogenin: novel 40 kDa fragment assigned to its N terminus. J Exp Biol 214: 582–592. 21270306 10.1242/jeb.048314 PMC3027471

[31] Li J, Huang J, Cai W, Zhao Z, Peng W, Wu J (2010) The vitellogenin of the bumblebee, Bombus hypocrita: studies on structural analysis of the cDNA and expression of the mRNA. J Comp Physiol B 180: 161–170. 20012056 10.1007/s00360-009-0434-5

[32] Mann CJ, Anderson TA, Read J, Chester SA, Harrison GB, et al (1999) The structure of vitellogenin provides a molecular model for the assembly and secretion of atherogenic lipoproteins. J Mol Biol 285: 391–408. 9878414 10.1006/jmbi.1998.2298

[33] Engelmann F (1977) Undegraded vitellogenin polysomes from female insect fat bodies. Biochem Biophys Res Commun 78: 641–647. 907702 10.1016/0006-291x(77)90227-3

[34] Raikhel AS, Dhadialla TS (1992) Accumulation of yolk proteins in insect oocytes. Annu Rev Entomol 37: 217–251. 1311540 10.1146/annurev.en.37.010192.001245

[35] Wheeler DE, Kawooya JK (1990) Purification and characterization of honey bee vitellogenin. Arch Insect Biochem Physiol 14: 253–267. 2134180 10.1002/arch.940140405

[36] Barchuk AR, Bitondi MM, Simoes ZL (2002) Effects of juvenile hormone and ecdysone on the timing of vitellogenin appearance in hemolymph of queen and worker pupae of Apis mellifera. J Insect Sci 2: 1. 15455035 10.1673/031.002.0101 PMC355901

[37] Engels W, Kaatz H, Zillikens A, Simoes ZLP, Trube A, et al.. (1990) Honey bee reproduction: vitellogenin and caste-specificregulation of fertility. Amsterdam: Elsevier.

[38] Spieth J, Nettleton M, Zucker-Aprison E, Lea K, Blumenthal T (1991) Vitellogenin motifs conserved in nematodes and vertebrates. J Mol Evol 32: 429–438. 1904098 10.1007/BF02101283

[39] Amdam GV, Simoes ZL, Guidugli KR, Norberg K, Omholt SW (2003) Disruption of vitellogenin gene function in adult honeybees by intra-abdominal injection of double-stranded RNA. BMC Biotechnol 3: 1. 12546706 10.1186/1472-6750-3-1 PMC149360

[40] Amdam GV, Simoes ZL, Hagen A, Norberg K, Schroder K, et al (2004) Hormonal control of the yolk precursor vitellogenin regulates immune function and longevity in honeybees. Exp Gerontol 39: 767–773. 15130671 10.1016/j.exger.2004.02.010

[41] Guidugli KR, Nascimento AM, Amdam GV, Barchuk AR, Omholt S, et al (2005) Vitellogenin regulates hormonal dynamics in the worker caste of a eusocial insect. FEBS Lett 579: 4961–4965. 16122739 10.1016/j.febslet.2005.07.085

[42] Nelson CM, Ihle KE, Fondrk MK, Page RE, Amdam GV (2007) The gene vitellogenin has multiple coordinating effects on social organization. PLoS Biol 5: e62. 17341131 10.1371/journal.pbio.0050062 PMC1808115

[43] Seehuus SC, Norberg K, Gimsa U, Krekling T, Amdam GV (2006) Reproductive protein protects functionally sterile honey bee workers from oxidative stress. Proc Natl Acad Sci U S A 103: 962–967. 16418279 10.1073/pnas.0502681103 PMC1347965

[44] Chen JS, Sappington TW, Raikhel AS (1997) Extensive sequence conservation among insect, nematode, and vertebrate vitellogenins reveals ancient common ancestry. J Mol Evol 44: 440–451. 9089084 10.1007/pl00006164

[45] Sappington TW, Raikhel AS (1998) Molecular characteristics of insect vitellogenins and vitellogenin receptors. Insect Biochem Mol Biol 28: 277–300. 9692232 10.1016/s0965-1748(97)00110-0

[46] Aalberse RC, Akkerdaas J, van Ree R (2001) Cross-reactivity of IgE antibodies to allergens. Allergy 56: 478–490. 11421891 10.1034/j.1398-9995.2001.056006478.x

[47] Hemmer W, Focke M, Kolarich D, Dalik I, Gotz M, et al (2004) Identification by immunoblot of venom glycoproteins displaying immunoglobulin E-binding N-glycans as cross-reactive allergens in honeybee and yellow jacket venom. Clin Exp Allergy 34: 460–469. 15005742 10.1111/j.1365-2222.2004.01897.x

[48] Jappe U, Raulf-Heimsoth M, Hoffmann M, Burow G, Hubsch-Muller C, et al (2006) In vitro hymenoptera venom allergy diagnosis: improved by screening for cross-reactive carbohydrate determinants and reciprocal inhibition. Allergy 61: 1220–1229. 16942573 10.1111/j.1398-9995.2006.01232.x

[49] Fujikawa A, Ishimaru N, Seto A, Yamada H, Aki T, et al (1996) Cloning and characterization of a new allergen, Mag 3, from the house dust mite, Dermatophagoides farinae: cross-reactivity with high-molecular-weight allergen. Mol Immunol 33: 311–319. 8649452 10.1016/0161-5890(95)00127-1

[50] Epton MJ, Dilworth RJ, Smith W, Thomas WR (2001) Sensitisation to the lipid-binding apolipophorin allergen Der p 14 and the peptide Mag-1. Int Arch Allergy Immunol 124: 57–60. 11306926 10.1159/000053668

[51] Epton MJ, Dilworth RJ, Smith W, Hart BJ, Thomas WR (1999) High-molecular-weight allergens of the house dust mite: an apolipophorin-like cDNA has sequence identity with the major M-177 allergen and the IgE-binding peptide fragments Mag1 and Mag3. Int Arch Allergy Immunol 120: 185–191. 10592463 10.1159/000024266

[52] Shimizu Y, Nakamura A, Kishimura H, Hara A, Watanabe K, et al (2009) Major allergen and its IgE cross-reactivity among salmonid fish roe allergy. J Agric Food Chem 57: 2314–2319. 19226142 10.1021/jf8031759

[53] Perez-Gordo M, Sanchez-Garcia S, Cases B, Pastor C, Vivanco F, et al (2008) Identification of vitellogenin as an allergen in Beluga caviar allergy. Allergy 63: 479–480. 18315737 10.1111/j.1398-9995.2007.01614.x

[54] Amo A, Rodriguez-Perez R, Blanco J, Villota J, Juste S, et al (2010) Gal d 6 is the second allergen characterized from egg yolk. J Agric Food Chem 58: 7453–7457. 20509661 10.1021/jf101403h

[55] King TP, Joslyn A, Kochoumian L (1985) Antigenic cross-reactivity of venom proteins from hornets, wasps, and yellow jackets. J Allergy Clin Immunol 75: 621–628. 3989148 10.1016/0091-6749(85)90040-5

[56] Jin C, Focke M, Leonard R, Jarisch R, Altmann F, et al.. (2010) Reassessing the role of hyaluronidase in yellow jacket venom allergy. J Allergy Clin Immunol 125: 184–190 e181. 10.1016/j.jaci.2009.08.037 19910026

