# Supplementary material for: Vitellogenins Are New High Molecular Weight Components and Allergens (Api m 12 and Ves v 6) of Apis mellifera and Vespula vulgaris Venom
Source: PLoS One. 2013 Apr 23;8(4):e62009. doi: 10.1371/journal.pone.0062009 (PMC3633918; doi:10.1371/journal.pone.0062009)

**Supplemental data figure S1: Immunoreactivity of Api m 12 with pooled sera of honeybee venom allergic patients.**

## Purified Api m 12 was separated by SDS-PAGE and immobilized onto a nitrocellulose membrane. Sera from 4 patients who showed specific IgE reactivity in ELISA (patients 16, 18, 24, and 29 in figure 5A) were pooled and diluted 1:10 with 5mg/ml BSA in PBS and applied to the Western blot. Visualization of bound IgE was then performed with anti-human IgE mAb conjugated to alkaline phosphatase and nitrotetrazolium blue chloride/5-bromo-4-chloro-3-indoyl phosphate.


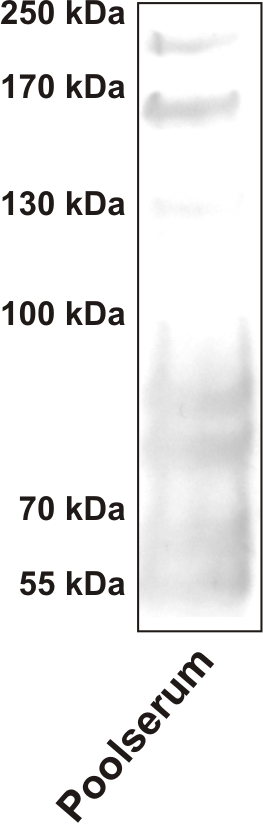

Supplement: Figure S1 — Immunoreactivity of Api m 12 with pooled sera of honeybee venom allergic patients. Purified Api m 12 was separated by SDS-PAGE and immobilized onto a nitrocellulose membrane. Sera from 4 patients who showed specific IgE reactivity in ELISA (patients 16, 18, 24, and 29 in figure 5A) were pooled and diluted 1∶10 with 5 mg/ml BSA in PBS and applied to the Western blot. Visualization of bound IgE was then performed with anti-human IgE mAb conjugated to alkaline phosphatase and nitrotetrazolium blue chloride/5-bromo-4-chloro-3-indoyl phosphate. (DOC) [file pone.0062009.s001.doc]
