# Supplementary material for: Vitellogenins Are New High Molecular Weight Components and Allergens (Api m 12 and Ves v 6) of Apis mellifera and Vespula vulgaris Venom
Source: PLoS One. 2013 Apr 23;8(4):e62009. doi: 10.1371/journal.pone.0062009 (PMC3633918; doi:10.1371/journal.pone.0062009)
Supplement: Table S1 — Serological data of patients assessed in IgE reactivity analysis. The sIgE levels for honeybee venom (HBV) (i1) and yellow jacket venom (YJV) (i3) were determined with the Immulite 2000 (Siemens Healthcare Diagnostics, Los Angeles, Ca.) or ImmunoCap 250 (Phadia, Uppsala, Sweden), and for Api m 12 and Ves v 6 as described for Fig. 5 (the lower end functional cutoff of the Api m 12 and Ves v 6 ELISA was OD405 = 0,55 and OD405 = 0,4, respectively). For intradermal testing of patients with suspected insect venom allergies serial 10-fold dilutions of venom extracts with concentrations ranging from 0.0001 to 0.1 mg/L were performed. Histamine hydrochloride and physiologic saline were used as positive and negative control solutions, respectively. Intradermal tests were rated positive when the wheal size was >5 mm in diameter with a surrounding erythema. (DOC) [file pone.0062009.s002.doc]

**Supplemental data table S1: Serological data of patients assessed in IgE reactivity analysis**

The sIgE levels for honeybee venom (HBV) (i1) and yellow jacket venom (YJV) (i3) were determined with the Immulite 2000 (Siemens Healthcare Diagnostics, Los Angeles, Ca.) or ImmunoCap 250 (Phadia, Uppsala, Sweden), and for Api m 12 and Ves v 6 as described for Fig. 5 (the lower end functional cutoff of the Api m 12 and Ves v 6 ELISA was OD_405_ = 0,55 and OD_405_ = 0,4, respectively). For intradermal testing of patients with suspected insect venom allergies serial 10-fold dilutions of venom extracts with concentrations ranging from 0.0001 to 0.1 mg/L were performed. Histamine hydrochloride and physiologic saline were used as positive and negative control solutions, respectively. Intradermal tests were rated positive when the wheal size was >5 mm in diameter with a surrounding erythema.

**Table 1.1 Serological data of patients assessed in IgE reactivity analysis with Api m 12**

| Patient  I.D. | Skin test^1^ | | sIgE – Extract^2^ | | sIgE – Allergen^3^ |
| --- | --- | --- | --- | --- | --- |
|  | HBV  (µg/ml) | ­­VV  (µg/ml) | i1  (kU/L) | i3  (kU/L) | Api m 12  (OD_405nm_) |
| 1 | 0,01 | 0,001 | 3,15 | 4,46 | 0,3112 |
| 2 | 0,01 | 0,001 | 32,9 | 14,4 | 0,4184 |
| 3 | 0,0001 | 0,1 | 9,48 | 0,33 | 0,5232 |
| 4 | 0,1 | 0,0001 | 1,76 | 16,4 | 0,2725 |
| 5 | 0,01 | 0,01 | 1,09 | 35,1 | 0,9419 |
| 6 | 0,0001 | 0,01 | 38,7 | 59,7 | 2,9238 |
| 7 | 0,01 | negative | >100 | 6,72 | 0,4292 |
| 8 | 0,01 | negative | 0.62 | 0.63 | 0,3078 |
| 9 | 0,1 | 0,0001 | 14,1 | 20 | 0,6766 |
| 10 | 0,0001 | 0,001 | 13,8 | 17,8 | 2,8877 |
| 11 | 0,01 | negative | 0,62 | 0,63 | 0,3914 |
| 12 | 0,1 | 0,01 | 5,94 | 2,14 | 0,3839 |
| 13 | 0,0001 | 0,01 | >100 | 0,472 | 0,6847 |
| 14 | 0,001 | 0,0001 | 1,44 | 6,04 | 0,8248 |
| 15 | 0,01 | 0,001 | 25,9 | >100 | 0,4172 |
| 16 | 0,1 | 0,01 | 0,163 | 4,34 | 2,1164 |
| 17 | 0,0001 | 0,001 | 1,47 | 7,060 | 0,593 |
| 18 | 0,1 | 0,01 | <0,1 | 0,775 | 3,539 |
| 19 | 0,001 | negative | 13,4 | 1,58 | 0,3845 |
| 20 | 0.001 | 0.01 | 0,436 | 0,191 | 0,3764 |
| 21 | 0,0001 | negative | 60.8 | 0.521 | 0,2896 |
| 22 | 0,001 | negative | 0,76 | 0,15 | 0,5238 |
| 23 | 0,001 | negative | 27,6 | <0.1 | 0,3811 |
| 24 | 0,01 | negative | 6,59 | 0.224 | 1,752 |
| 25 | 0,1 | negative | 2,33 | <0,1 | 0,4364 |
| 26 | 0,001 | 0,1 | 10,2 | 14,5 | 0,389 |
| 27 | 0,0001 | negative | 23,8 | 2,19 | 0,4201 |
| 28 | 0,0001 | negative | 72,7 | 0,124 | 0,3515 |
| 29 | 0,01 | 0,1 | 3,21 | 2,17 | 2,5601 |
| 30 | 0,001 | negative | 35,9 | 0,889 | 0,6065 |
| 31 | 0,01 | negative | 81,4 | 3,31 | 3,4815 |
| 32 | 0,0001 | 0,01 | 1,89 | 0,19 | 0,8964 |
| 33 | 0,01 | 0,01 | 4,32 | 8,31 | 2,4411 |
| 34 | 0,0001 | negative | 3,26 | 0,11 | 0,6912 |
| 35 | 0,001 | 0,001 | 1,33 | 0.467 | 0,5213 |
| 36 | 0,01 | 0,01 | 3,88 | 0,4 | 0,9023 |
| 37^4^ | nd | nd | 20,5 | 1,42 | 0,4234 |
| 38^5^ | nd | nd | 3,54 | 6,75 | 0,3664 |
| 39 | 0,01 | 0,0001 | 5,200 | 17,900 | 0,7892 |
| 40^4^ | nd | nd | 13,800 | 8,290 | 0,4692 |
| 41 | 0,1 | negative | 0,963 | <0,1 | 3,2868 |
| 42 | nd | nd | 22,2 | 2,82 | 0,5253 |
| 43 | 0,01 | 0,01 | 5,46 | 3,64 | 1,136 |
| 44 | 0,0001 | 0,01 | 4,01 | 0,226 | 0,634 |
| 45 | 0,01 | negative | 14,1 | 0,81 | 0,4517 |

**Table 1.2 Serological data of patients assessed in IgE reactivity analysis with Ves v 6**

| Patient  I.D. | Skin test^1^ | | sIgE – Extract^2^ | | sIgE – Allergens^3^ |
| --- | --- | --- | --- | --- | --- |
|  | HBV  (µg/ml) | ­­VV  (µg/ml) | i1  (kU/L) | i3  (kU/L) | Ves v 6  (OD_405nm_) |
| 1 | negative | 0,0001 | <0,1 | >100 | 0,3943 |
| 2 | 0,01 | 0,001 | 3,15 | 4,46 | 0,3063 |
| 3 | negative | 0,001 | <0,1 | 2,0 | 1,5938 |
| 4 | 0,01 | 0,001 | 32,9 | 14,4 | 0,8111 |
| 5 | 0,1 | 0,01 | <0,1 | 0,775 | 0,3156 |
| 6 | negative | 0,001 | 0,15 | 10,8 | 0,3341 |
| 7 | negative | 0,01 | 1,16 | 19,0 | 0,6781 |
| 8 | 0,1 | 0,0001 | 1,76 | 16,4 | 0,2913 |
| 9 | 0,01 | 0,01 | 1,09 | 35,1 | 0,7891 |
| 10 | 0,0001 | 0,001 | 1,47 | 7,060 | 0,3387 |
| 11 | 0,0001 | 0,01 | 38,7 | 59,7 | 1,4818 |
| 12 | 0,01 | 0,01 | 5,46 | 3,64 | 0,3897 |
| 13 | negative | 0,001 | 0,37 | 1,59 | 1,3225 |
| 14 | 0,1 | 0,0001 | 14,1 | 20 | 0,3590 |
| 15 | 0,0001 | 0,001 | 13,8 | 17,8 | 1,3320 |
| 16 | 0,1 | 0,01 | 5,94 | 2,14 | 0,8710 |
| 17 | 0,0001 | 0,01 | >100 | 0,472 | 0,2941 |
| 18 | 0,001 | 0,0001 | 1,44 | 6,04 | 0,7146 |
| 19 | 0,01 | 0,001 | 25,9 | >100 | 0,3271 |
| 20 | 0,1 | 0,01 | 0,163 | 4,34 | 0,3222 |
| 21 | negative | 0,1 | <0,1 | 1,1 | 0,3398 |
| 22 | 0,1 | 0,01 | <0,1 | 4,67 | 0,2639 |
| 23 | negative | 0,0001 | 0,568 | >100 | 1,4531 |
| 24 | negative | 0,001 | 0,12 | 8,18 | 0,3095 |
| 25 | negative | 0,01 | <0,1 | 24 | 0,2617 |
| 26 | nd | nd | <0,1 | 8,69 | 0,2890 |
| 27 | 0,0001 | 0,1 | 9,48 | 0,33 | 1,2284 |
| 28 | nd | nd | <0,1 | 1,81 | 0,3201 |

^1^ Shown is the lowest venom extract concentration that gave a positive result in intradermal skin testing applying a ten-fold concentration range from 0.1 to 0.0001 µg/ml for each of the venoms.

^2^ As tested in either UniCAP250 or Immulite2000 sIgE assays using a lower end cut-off of 0.1 kU/L.

^3^ As tested in an IgE ELISA using a lower end cut-off of 0.55 for Api m 12 and of 0.4 for Ves v 6, respectively, as described in the methods section.

^4^ No skin test was performed, but these patients were medicated in the casualty department due to a clear clinical reaction after a honeybee sting.

^5^ Due to a clinical history of Urticaria factitia for this patient the skin test is not evaluable.

nd, not determined
